# Deletions of the *SACPD-C* locus elevate seed stearic acid levels but also result in fatty acid and morphological alterations in nitrogen fixing nodules

**DOI:** 10.1186/1471-2229-14-143

**Published:** 2014-05-27

**Authors:** Jason D Gillman, Minviluz G Stacey, Yaya Cui, Howard R Berg, Gary Stacey

**Affiliations:** 1 USDA-ARS, University of Missouri-Columbia, 205 Curtis Hall, Columbia MO 65211, USA; 2 Divisions of Plant Science and Biochemistry, University of Missouri-Columbia, Columbia MO 65211, USA; 3 Donald Danforth Plant Science Center, St. Louis MO, USA

**Keywords:** Soybean (*Glycine max*), Stearic acid, Fatty acid composition, Radiation mutagenesis, Comparative genome hybridization, Nodulation

## Abstract

**Background:**

Soybean (*Glycine max*) seeds are the primary source of edible oil in the United States. Despite its widespread utility, soybean oil is oxidatively unstable. Until recently, the majority of soybean oil underwent chemical hydrogenation, a process which also generates *trans* fats. An alternative to chemical hydrogenation is genetic modification of seed oil through identification and introgression of mutant alleles. One target for improvement is the elevation of a saturated fat with no negative cardiovascular impacts, stearic acid, which typically constitutes a minute portion of seed oil (~3%).

**Results:**

We examined radiation induced soybean mutants with moderately increased stearic acid (10-15% of seed oil, ~3-5 X the levels in wild-type soybean seeds) via comparative whole genome hybridization and genetic analysis. The deletion of one *SACPD* isoform encoding gene (*SACPD-C*) was perfectly correlated with moderate elevation of seed stearic acid content. However, *SACPD-C* deletion lines were also found to have altered nodule fatty acid composition and grossly altered morphology. Despite these defects, overall nodule accumulation and nitrogen fixation were unaffected, at least under laboratory conditions.

**Conclusions:**

Although no yield penalty has been reported for moderate elevated seed stearic acid content in soybean seeds, our results demonstrate that genetic alteration of seed traits can have unforeseen pleiotropic consequences. We have identified a role for fatty acid biosynthesis, and SACPD activity in particular, in the establishment and maintenance of symbiotic nitrogen fixation.

## Background

Soybean (*Glycine max* (L.) Merr) seed oil is the most widely utilized edible oil in the United States (~66% of total edible fats), and the second most widely consumed edible oil worldwide (~28%). The majority (94%) of US soybean oil is used for salad/cooking, frying/baking and industrial uses, representing ~53%, ~21%, and 20% respectively (http://soystats.com/archives/2012/non-frames.htm, compiled from USDA statistics).

Until very recently the majority of soybean oil underwent partial or full hydrogenation to increase oxidative stability [1]. This practice also generates *trans* fats, which has attracted negative public attention due to the findings that high dietary intake of *trans* fats elevated blood serum levels of low density lipoprotein (LDL) cholesterol [2] and elevated serum LDL levels are directly correlated with increased risk of coronary heart disease [3]. As a result, labeling of products containing *trans* fats is required by law within the United States [1] and the American Heart Association has recommended that *trans* fats be reduced as much as feasible (http://www.americanheart.org/).

Stearic acid (C18:0) is the desired end product of full hydrogenation of soybean oil (fully hydrogenated oils do not contain *trans* fats yet would likely be regulated similar to partially hydrogenated oils) and stearic acid has been shown to neither elevate nor reduce blood serum LDL cholesterol [2]. In controlled diets, the replacement of other saturated fats (such as palmitic acid) with “heart neutral” stearic acid was shown to be beneficial on LDL cholesterol levels [4]. Regrettably, stearic acid forms a minute portion of the total seed oil for most plants; only 3-4% of soybean seed oil is present as stearic acid in typical cultivars [5]. *Theobroma cacao* (chocolate) seeds possess an exceptional ~36.6% stearic acid content, which is used to make cocoa butter [6], but *T. cacao* is a rare exception and the potential for enhancing production of this tropical tree crop is extremely limited.

In Arabidopsis, loss of function for one specific (*fatb/ssi2*) *Stearoyl-Acyl Carrier Protein Desaturase (SACPD)* gene isoform increases both seed [7] and leaf stearic acid content [8], but also has pleiotropic effects on plant defense signaling [9]. Studies in Arabidopsis identified at least seven distinct isoforms that are expressed in various tissues. These isoforms were demonstrated to have activity differences for either C16:0 or C18:0 precursors [10]. In contrast to Arabidopsis, soybean has a smaller subset of SACPD gene isoforms, with only three actively transcribed (*SACPD-A*, Glyma07g32850; *SACPD-B*, Glyma02g15600; *SACPD-C*, Glyma14g27990). SACPD-A and –B protein products are highly similar (98% identity) and are predicted to be targeted to plastids [11]. SACPD-C is quite divergent from the other two SACPD isoforms (~63% identity with either SAPCD-A or –B) and it is not clear if SACPD-C protein is targeted solely to plastids or is dual targeted to plastids and mitochondria *in planta*[12].

Mutant soybean lines with elevated seed stearic acid content were first reported in the 1980’s. One sodium azide induced mutant line, A6, has a remarkable ~28% of the total seed oil present in the form of stearic acid (~8 to 10 fold higher than conventional soybeans) [13,14]. The increase in stearic acid content of seeds in A6 [13,14] was reported to be due solely to deletion of *SACPD-C*[12]. Unfortunately, a significant negative correlation was found between elevated stearic acid content and seed yield using the A6 mutant line. Additional mutant sources with slightly less stearic acid content (~11 to 15%) do not have the same negative association with seed yield [15].

In this work, we utilized CGH with four radiation induced mutant soybean lines with moderately elevated seed stearic acid (10 to 15%). The complimentary methods of CGH and genetic analysis were used to identify and confirm that the genetic basis for the moderately elevated seed stearic acid phenotype was due to mutations affecting the *SACPD-C* gene, in five independent mutant lines from multiple genetic backgrounds and mutagens. The *SACPD-C* gene is strongly expressed in seeds but also in nodules. In all of the independent mutant lines with elevated seed stearic acid, *SACPD-C* mutations also resulted in nodules with very atypical nodule structure. Under laboratory growth conditions, however, these changes did not affect nitrogen fixation levels.

## Results

### Oil phenotypes of mutant lines with elevated seed stearic acid

**Figure 1:**
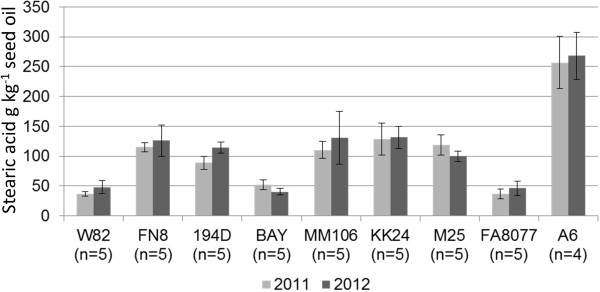
**Stearic acid seed phenotypes of selected radiation and EMS induced soybean mutant lines.** Height of histograms indicates mean seed stearic acid content from selected radiation and EMS induced soybean mutant lines compared to their progenitors (n = 4 or 5), produced at Columbia, MO (Bradford experiment field) in Summer 2011 or Summer 2012. Bars indicated one standard deviation above/below the mean.

The mutant lines KK24, MM106 and M25 were previously identified by a forward screen of X-ray induced mutant lines [16,17] of the soybean cultivar ‘Bay’ [18]. None of these three mutant lines (MM106, KK24, M25) were significantly different in seed stearic acid content when grown in either 2011 or 2012 (Figure 1, Table 1) at a Columbia, MO field location. A6 was released in 1983 as a sodium azide induced mutant of ‘FA8077’ and was reported to have an 8 to 10-fold increase in seed stearic acid levels (~28% of total oil) [14]. When grown in Columbia, MO, A6 was found to have 257 ± 44 g stearic acid kg^−1^seed oil in 2011 and similar levels (268 ± 39 g stearic acid kg^−1^seed oil) when grown in 2012. Full details on fatty acid profiles of these mutants are provided in Table 1.

**Table 1:** Seed fatty acid profile data for selected soybean lines grown in Columbia, MO field location

**Columbia, MO 2011**			**g kg-1 seed oil**^**1**^					
	**Mutagen**	***SACPD-C***	**n=**	**16:0**	**18:0**	**18:1**	**18:2**	**18:3**
**‘W82’**	-	WT	5	105 ± 8 A	37 ± 4 A	202 ± 17 AB	573 ± 20 A	86 ± 6 A
**194D**	FN	V211E	5	98 ± 3 AB	89 ± 11 B	189 ± 7 AB	546 ± 9 AB	78 ± 6 A
**FN8**	FN	ΔSACPD-C	5	91 ± 6 BC	115 ± 8 B	173 ± 4 A	538 ± 6 AB	83 ± 5 AB
**‘Bay’**	-	WT	5	99 ± 7 AB	52 ± 9 A	232 ± 14 AB	539 ± 14AB	77 ± 2 AB
**MM106**	X-ray	ΔSACPD-C	5	99 ± 4 AB	110 ± 14 B	182 ± 9 AB	522 ± 12 B	87 ± 5 A
**KK24**	X-ray	C298Δ	5	92 ± 8 ABC	129 ± 27 B	162 ± 15 A	537 ± 1 AB	80 ± 2 AB
**M25**	X-ray	C298Δ	5	101 ± 5 AB	119 ± 17 B	169 ± 15 A	524 ± 17 B	87 ± 2 A
**‘FA8077’**	-	WT	5	93 ± 8 ABC	37 ± 7 A	341 ± 66 C	460 ± 46 C	69 ± 7 B
**A6**	unclear	ΔSACPD-C	4	82 ± 7 C	257 ± 44 C	176 ± 36 A	404 ± 36 D	82 ± 9 A
**Columbia, MO 2012**			**g kg-1 seed oil**^**1**^					
	**Mutagen**	**SACPD-C**	**n=**	**16:0**	**18:0**	**18:1**	**18:2**	**18:3**
**‘W82’**	-	WT	5	106 ± 10 A	48 ± 11 A	230 ± 26 A	547 ± 19 A	70 ± 6 AB
**194D**	EMS	V211E	5	95 ± 8 A	114 ± 10 B	199 ± 27 A	514 ± 31AB	78 ± 8 ABC
**FN8**	FN	ΔSACPD-C	5	91 ± 4 A	126 ± 26 B	165 ± 7 A	540 ± 19 A	78 ± 5 ABC
**‘Bay’**	-	WT	5	107 ± 9 A	40 ± 6 A	207 ± 30 A	556 ± 30 A	90 ± 11 C
**MM106**	X-ray	ΔSACPD-C	5	96 ± 8 A	131 ± 45 B	173 ± 13 A	518 ± 27 AB	82 ± 4 BC
**KK24**	X-ray	C298Δ	5	90 ± 5 A	131 ± 19 B	162 ± 11 A	539 ± 25 A	77 ± 6 ABC
**M25**	X-ray	C298Δ	5	106 ± 6 A	100 ± 9 B	159 ± 7 A	547 ± 13 A	89 ± 4 C
**‘FA8077’**	-	WT	5	101 ± 7 A	46 ± 12 A	327 ± 81 B	464 ± 70 B	62 ± 14 A
**A6**	Unclear	ΔSACPD-C	4	92 ± 16 A	268 ± 39 C	218 ± 32 A	358 ± 46 C	65 ± 17 AB

^1^Letters adjacent to mean + standard deviation indicates result of Tukey’s HSD test (α = 0.05); common letters indicate insignificant differences between means.

### Comparative genome hybridization and sequence analysis of mutant line MM106

Radiation induced mutagenesis can result in genomic deletions, which can vary in size from single base deletions/alterations to chromosomal level deletions, translocations and inversions [19]. Comparative Genomic Hybridization (CGH) using microarray slides has emerged as a powerful tool to quantify genomic deletions and copy number variants [20]. We utilized a custom soybean CGH array [21], based on the ‘Williams 82’ genome sequence [22], to compare the mutant line MM106 with its progenitor line ‘Bay’. Based on previous work which demonstrated that deletion of the *SACPD-C* locus in line A6 elevated seed stearic acid to ~28% [12], we anticipated that MM106 bore a deletion(s) distinct from *SACPD-C*.

**Figure 2:**
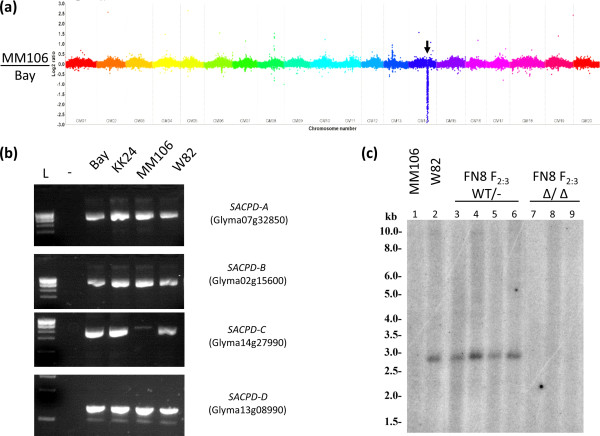
**Comparative Genome Hybridization analysis of MM106 in comparison to ‘Bay’. (a)** Entire genome view of Comparative Genome Hybridization for MM106/’Bay’. Deletion affecting Gm14 is indicated by arrow. **(b)** PCR assay for detecting *stearoyl-acyl carrier protein desaturase (SACPD)* gene deletions from ‘Bay’ radiation induced mutant lines. L indicate molecular weight ladder, “-“ indicates negative control. **(c)** Southern blot analysis with a *SACPD-C* specific probe against DNA from MM106 (1), ‘Williams 82’(2), and FN8 F_2:3_ segregants which displayed typical levels of seed stearic acid (4–6) and FN8 F_2:3_ samples which displayed elevated levels of seed stearic acid (7–9).

**Figure 3:**
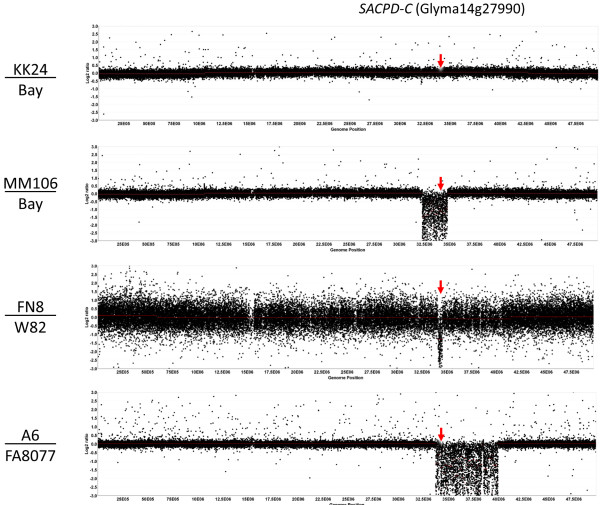
**Comparative Genome Hybridization output for chromosome 14 for KK24/’Bay’, MM106/’Bay’, FN8/’Williams 82’, and A6/’FA8077’.** The approximate position of *SACPD-C* gene locus (Glyma14g27990) is indicated by the arrows.

The CGH technique revealed a moderately large deletion (~2.5 Mbp) affecting chromosome 14 in MM106 (Figure 2a, Table 2). In contrast to our *a priori* expectations, the larger deletion was found to include the *SACPD-C* locus (Figure 2b, c and Figure 3), 30 additional genes from the Glyma 1.0 high confidence gene set (and a portion of another 2 genes), and 47 genes from the “low confidence” gene set (ftp://ftp.jgi-psf.org/pub/compgen/phytozome/v9.0/Gmax/). Despite attempts with 10 different primer pairs, all efforts to bridge this deletion were unsuccessful (data not shown). However, the absence of *SACPD-C* was confirmed by PCR (Figure 2b) and by Southern blot analysis (Figure 2c).

**Table 2:** Statistically significant deletions identified in elevated stearic acid mutant lines by Comparative Genome Hybridization technique

**CGH comparison**	**Fold ratio (neg = deletion)**	**CHR**	**~ deletion start**	**~ deletion end**	**Deletion size (bps)**	**% CHR affected**	***SACPD-C***	**Glyma1 gene models affected**
FN8/W82	−1.08	Gm14	34,116,388	34,523,951	407,563	0.82%	*ΔSACPD-C*	12
MM106/Bay	−0.83	Gm11	10,333,512	10,335,676	2,164	0.01%		1 (partial)
	−1.71	Gm14	32,288,267	34,779,838	2,491,571	5.01%	*ΔSACPD-C*	80 (2 partial)
	−1.84	Gm18	4,557,847	4,560,113	2,266	0.00%		None
	−1.13	Gm18	4,582,012	4,584,221	2,209	0.00%		1 (partial, low confidence)
KK24or M25/Bay	−2.48	Gm11	4,376,963	4,559,394	182,431	0.47%		26
	−0.65	Gm11	10,329,096	10,359,964	30,868	0.08%		3 + 2 (partial)
	N/A	Gm14	34324782	34324782	1	<.0001%	C298Δ	Glyma14g27990
A6/FA8077	−1.43	Gm02	4,858,850	4,863,246	4,396	0.01%		
	−2.32	Gm02	40,861,581	41,125,894	264,313	0.51%		
	−0.75	Gm09	8,937,856	8,940,059	2,203	0.00%		None
	−0.49	Gm10	6,316,784	6,325,045	8,261	0.02%		1 (partial)
	−0.77	Gm10	39,664,757	39,689,162	24,405	0.05%		4
	−0.96	Gm10	40,573,756	40,575,906	2,150	0.00%		None
	−0.67	Gm10	40,672,171	40,679,285	7,114	0.01%		None
	−1.74	Gm10	41,199,204	41,201,464	2,260	0.00%		1 (partial)
	−1.29	Gm11	37,009,044	37,029,501	20,457	0.05%		4
	−1.39	Gm14	33,716,793	39,937,769	6,220,976	12.51%	*ΔSACPD-C*	143
	−0.59	Gm15	19,611,579	19,640,675	29,096	0.06%		1 + 1 (partial)

Two additional small deletions affecting separate chromosomes are predicted to result in partial deletions of two gene models in MM106 (Glyma11g14490 and Glyma18g05970-low confidence gene set). We also noted several genomic regions which displayed increased probe signal, which could indicate the presence of a radiation induced duplication, herein termed a Copy Number Variant (CNV). A summary of all genomic deletions identified is provided in Table 2 and full details on statistically significant deletions and putative CNV are included in Additional file 1.

### CGH and Sanger sequencing analysis of M25 and KK24

**Figure 4:**
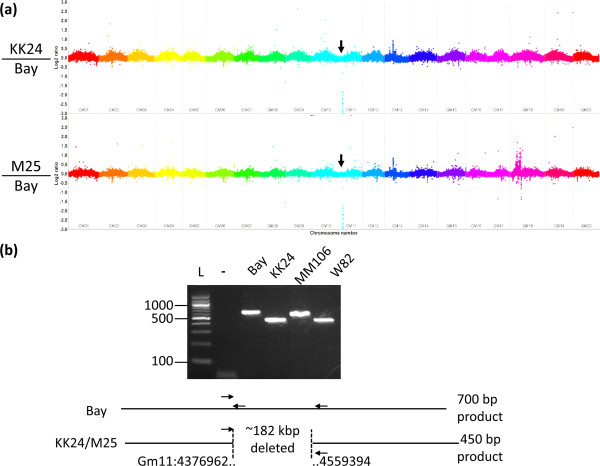
**Comparative Genome Hybridization analysis of KK24 and M25 in comparison to ‘Bay’. (a)** Entire genome view of Comparative Genome Hybridization for KK24/’Bay’ and M25/’Bay’. Deletion affecting Gm11 indicated by arrow. **(b)** PCR assay for detecting *stearoyl-acyl carrier protein desaturase (SACPD)* gene deletions. The position of primers is indicated by arrows. For homozygous wild type lines, a ~700 bp PCR product is produced, whereas for homozygous mutant lines, only a 450 bp product is generated.

We also utilized the CGH technique to compare two other ‘Bay’ derived high stearic lines, created during the same mutagenesis experiment [16,17]. We noted highly similar hybridization patterns for M25 and KK24 as compared to ‘Bay’ (Figure 4a) and both KK24 and M25 have a common ~182 kbps genomic deletion affecting chromosome 11 (Table 1). We utilized PCR to bridge this deletion (Figure 4b) and sequencing of the PCR product revealed that both lines bear an identical simple ~182 kbp genomic deletion (Table 1) with no extraneous DNA inserted. The common Gm11 deletion is predicted to result in loss of 25 genes from the high confidence Glyma 1.09 gene set, and another 42 from the low confidence list (Table 2).

For M25, the hybridization signal for probes corresponding to the proximal arm of chromosome 18 were highly variable (Figure 4a, Additional file 2). A similar variability was observed for certain genomic regions when comparing ‘Williams 82’ accessions from different seed stocks [21] and was attributed to residual heterozygosity in the original BC_6_F_2:3_ ‘Williams 82’ [23] line, prior to seed distribution. We examined several Simple Sequence Repeat (SSR) markers corresponding to this region for M25, KK24, MM106 and ‘Bay’ and noted polymorphism between M25 and KK24/Bay/MM106 (Additional file 2), which supports the hypothesis that the common ancestor of ‘Bay’/MM106/M25/KK24 bore residual heterozygosity in this region.

**Figure 5:**
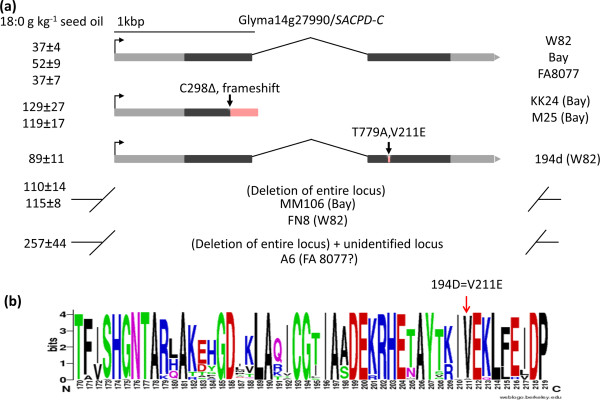
**Cartoon depiction of the *SACPD-C *(Glyma14g27990) locus in various radiation and EMS-induced mutant lines. (a)** An arrow indicates the predicted transcriptional start sites, dark gray boxes indicate exons, introns are indicated by lines, and 5’- or 3’- untranslated regions are represented by light gray boxes. Regions with amino acid sequence due to mutagenesis are indicated by red lines or blocks. **(b)** Weblogo SACPD-C amino acid sequence surrounding the residue mutated (V211E) in EMS induced high stearic acid mutant line 194D. The height of letters is proportionate to the degree of conservation among 100 diverse NCBI entries matching SACPD-C. The specific residue affecting by the 194D mutation is indicated by arrow. Image was created using online tool (http://weblogo.berkeley.edu/).

The CGH technique did not reveal any large deletions in the vicinity of any *SACPD* genes for M25/KK24 (Figure 3). However, small deletions could potentially be missed using the current array. To address this possibility, we also PCR amplified and Sanger sequenced each of the four known *SACPD* genes in soybean for M25, KK24 and ‘Bay’ (*SACPD-A*, Glyma07g32850; *SACPD-B*, Glyma02g15600; *SACPD-C*, Glyma14g27990; and a non-expressed pseudogene we termed *SACPD-D*, Glyma13g08990). Sequence traces for *SACPD-A*, −*B* and *–D* were identical to ‘Bay’. For *SACPD-C,* KK24 and M25 bear a common single base deletion within exon 1 of *SACPD-C* (NCBI KF670869, C298Δ relative to start codon), which results in the introduction of a frameshift mutation starting at codon 100 (Figure 5a).

Based on the highly similar overall CGH pattern, the identical single base deletion within exon 1 of *SACPD-C,* and the identical genomic deletion affecting Gm11, it is clear that M25 and KK24 arose from a single line. Despite this common origin, these lines are not identical. The most likely possibility is that the original ‘Bay’ seed that gave rise to M25/KK24 had residual heterozygosity for the Gm18 region that has since segregated in the progeny.

### Analysis of segregating F_2:3_ progeny from crossing MM106 or KK24 to wild type lines

A SimpleProbe based molecular marker assay was developed to track the single base deletion in KK24/M25 (Additional files 3 and 4). This allowed statistical analysis of phenotypic data points based on *SACPD-C* genotypic categories. Homozygosity for the single base deletion was found to be perfectly associated with moderately increased seed stearic acid content (Table 3).

**Table 3:** Seed fatty acid profile data for selected lines from crosses between moderately elevated stearic acid and wild type lines

			**g kg-1 seed oil**^**1**^						
**Line**	**Generation**	***SACPD-C***	**n=**	**Expect**	**16:0**	**18:0**	**18:1**	**18:2**	**18:3**
**FN8 x W82**	BC_1_F_2_	Δ/Δ	12	10	89 ± 3 A	133 ± 18 A	168 ± 5 A	530 ± 15 A	80 ± 5 A
		WT/_	28	30	104 ± 4 B	51 ± 10 B	230 ± 23 B	541 ± 18 A	74 ± 8 A
**FN8**	Pureline	Δ/Δ	4	n/a	91 ± 4 A	126 ± 26 A	165 ± 7 A	540 ± 19 A	78 ± 5 A
**W82**	Pureline	WT	4	n/a	103 ± 3 B	39 ± 3 B	200 ± 8 B	576 ± 9 A	82 ± 7 A
**MM106 X W82**	F_2:3_	Δ/Δ	4	4	112 ± 5 A	90 ± 13 A	171 ± 6 A	545 ± 7 AB	81 ± 8 A
		WT/_	15	16	129 ± 7 B	44 ± 6 C	197 ± 29 A	553 ± 18 AB	78 ± 9 A
**MM106**	Pureline	Δ/Δ	4	n/a	100 ± 8 A	114 ± 26 B	178 ± 21 A	532 ± 26 B	76 ± 12 A
**W82**	Pureline	WT	4	n/a	103 ± 3 A	39 ± 3 C	200 ± 8 A	576 ± 9 A	82 ± 7 A
**MM106 x KK24**	F_2:3_	Δ/Δ	18	16.5	100 ± 11 A	112 ± 28 A	198 ± 30 A	529 ± 33 A	61 ± 11 A
		C298Δ/_	4	5.5	97 ± 5 A	115 ± 9 A	209 ± 29 A	524 ± 34 A	56 ± 7 A
**MM106**	Pureline	Δ/Δ	4	n/a	100 ± 8 A	114 ± 26 A	178 ± 21 A	532 ± 26 A	76 ± 12 A
**KK24**	Pureline	C298Δ	4	n/a	102 ± 13 A	97 ± 37 A	167 ± 28 A	565 ± 31 A	70 ± 15 A
**KK24 X W82**	F_2:3_	C298Δ/C298Δ	9	10	105 ± 4 AB	89 ± 15 A	169 ± 14 A	564 ± 19 A	73 ± 5 A
		C298Δ/WT	20	20	113 ± 4 C	50 ± 8 B	184 ± 26 A	582 ± 16 AB	71 ± 8 A
		WT/WT	12	10	116 ± 4 C	37 ± 4 B	184 ± 26 A	597 ± 19 B	70 ± 7 A
**KK24**	Pureline	C298Δ	4	n/a	102 ± 13 A	97 ± 37 A	167 ± 28 A	565 ± 31 A	70 ± 15 A
**W82**	Pureline	WT	4	n/a	103 ± 3 BC	39 ± 3 B	200 ± 8 A	576 ± 9 AB	82 ± 7 A

^1^Letters adjacent to mean + standard deviation indicates result of Tukey’s HSD test (α = 0.05); common letters indicate insignificant differences between means.

Since it was not possible to bridge the deletion in MM106, we used PCR primers specific for the *SACPD-C* locus (Additional file 3) to detect homozygous mutants. It was not possible to differentiate heterozygotes from homozygote wild type lines using this method. Nevertheless, homozygosity for the *SACPD-C* deletion in MM106 was completely associated with elevated seed stearic acid levels (90 ± 13 g stearic acid kg^−1^ seed oil, Table 3).

K24, M25 and MM106 were phenotypically indistinguishable from the genomic deletion present in MM106 during both field years (Table 1). We also crossed the single base deletion line KK24 with the entire locus deletion line MM106 and found no statistically significant difference between any of the progeny of this cross (Table 3).

### Comparative Genome Hybridization of elevated stearic fast neutron induced mutant line FN8

As part of an existing reverse genetic study [24], a fast neutron induced mutant line was identified which bears a relatively small deletion (~408 kilobase pairs) predicted to contain the *SACPD-C* locus (Figure 3; Table 2). This line displayed elevated seed stearic acid (115 ± 8 stearic acid kg^−1^seed oil), which was statistically indistinguishable from the *SACPD-C* deletion line MM106, KK24 or M25 (Figure 1, Table 1).

We examined the association of this deletion with seed fatty acid profile in a small BC_1_F_2_ population. As with other *SACPD-C* mutants, homozygosity for this deletion, as determined by Southern Blot analysis (Figure 2c) or by PCR based assay (Table 3), was perfectly correlated with the elevation of seed stearic acid content (115 ± 8 g kg-1 seed oil, Table 3).

### Identification of EMS induced *SACPD-C* mutant line 194D

We also performed a forward genetic screen on an Ethyl MethaneSulphonate (EMS) induced mutant population of ‘Williams 82’ for alterations in fatty acid composition. One line demonstrated elevated stearic acid (89 ± 11 g stearic acid kg^−1^seed oil) and was selected for further analysis. A single SNP was identified within exon two of the *SACPD-C* gene (NCBI # KF670870, T779A, Figure 5a), which resulted in a missense substitution of an almost invariant residue (V211E, Figure 5b).

### CGH and DNA sequence analysis of *SACPD-C* deletion line A6

**Figure 6:**
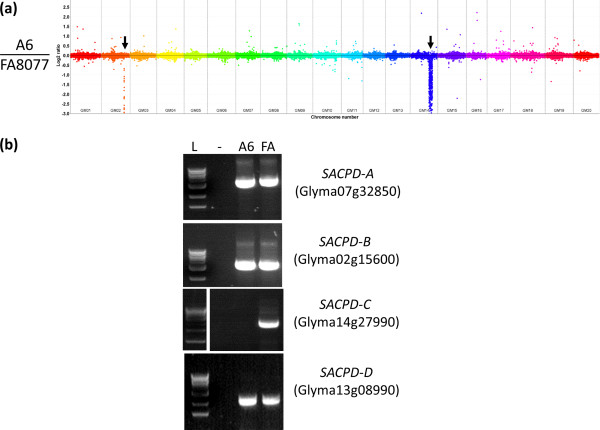
**Comparative Genome Hybridization analysis of A6 in comparison to ‘FA 8077’. (a)** Entire genome view of Comparative Genome Hybridization for A6/’FA 8077’. Large deletions affecting Gm02 and Gm14 are indicated by arrows. **(b)** PCR assay for detecting *Stearoyl-Acyl Carrier Protein Desaturase (SACPD)* gene deletion using A6 and ‘FA 8077’ (FA) gDNA.

The genomic deletion containing *SACPD-C* in A6 [12] was reported to have arisen due to sodium azide mutagenesis performed on seeds from ‘FA 8077’ [14]. However, the full extent of the genomic deletion has not been quantified. We utilized CGH to contrast A6 with the progenitor line ‘FA 8077’ (Figure 6). This revealed a range of small to medium deletions (<8 kbp to 29 kbp), a moderately large deleted region (~264 kbp) and one extraordinarily large deletion corresponding to ~1/8 of chromosome 14 (Table 2, Figure 6). The largest deletion identified (6221 kbp, ~12.5% of chromosome 14) contains the *SACPD-C* locus, as well as at least 56 genes from the high confidence gene set (and 87 presumed pseudogenes) as defined by the current Glyma 1.09 gene annotation (ftp://ftp.jgi-psf.org/pub/compgen/phytozome/v9.0/Gmax/). We also identified several overrepresented probe regions (CNVs, details are in Additional file 1).

### Analysis of *SACPD-A* and *–B* gene expression in A6

**Figure 7:**
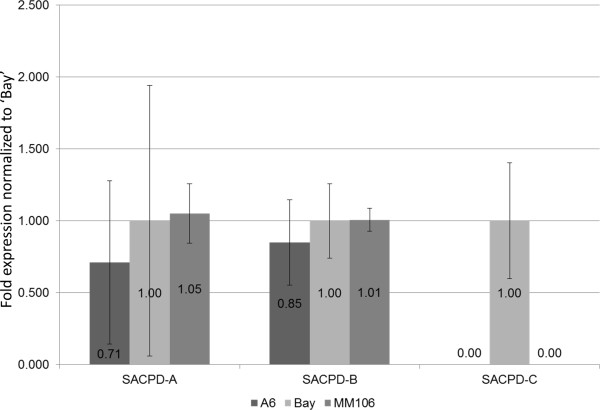
**Quantitative RT-PCR analysis of *Stearoyl-Acyl Carrier Protein Desaturase *** **(*SACPD) *** **genes using mRNA from mid-maturation seeds grown in Columbia, MO field location (Bradford experiment field) in 2012.**

One hypothesis for the difference in seed stearic acid content between the high stearic line A6 and the series of moderate stearic acid lines is that *SACPD-A* or *–B* could have impaired function. We amplified and Sanger sequenced these genes from ‘FA 8077’ and A6. For *SACPD-A* and *–D,* we observed no polymorphisms between A6 and ‘FA 8077’. We unexpectedly identified a large number of intronic and silent polymorphisms in *SACPD-B* (although none were predicted to affect the coding region) (Additional file 5). We also utilized quantitative RT-PCR to evaluate expression of *SACPD-A, −B* and *–C* during mid-maturation of green soybean seeds*.* The expression levels of *SACPD-A* and *–B* were not statistically different between any of the lines examined (Figure 7). In contrast, *SACPD-C* expression was completely absent in seeds from both MM106 and A6, as compared to ‘Bay’ (Figure 7).

### Analysis of nodule function and morphology in *SACPD-C* mutant lines

Soybean plants can establish a symbiotic interaction with certain soil bacteria (*e.g.*, *Bradyrhizobium japonicum*) which leads to the development of a new root organ, the nodule, where bacteria differentiate into bacteroids that fix atmospheric nitrogen for assimilation by the host plant. The ability of soybean to perform biological N_2_ fixation contributes to its agronomic importance and, on average accounts for 50-60% of soybean N requirement [25]. We utilized the publicly available genome-wide gene expression index for soybean [26,27] and the Soyseq resource (http://www.soybase.org/soyseq) to investigate the expression pattern of *SACPD*-related genes. We noted very high levels of expression of *SACPD-C* in both seeds and nodules (Additional file 6). Therefore, in addition to the effects of *SACPD-C* mutations on seed stearic acid levels, the potential exists for additional impacts on nodule development and physiology.

To determine if mutations in *SACPD-C* result in altered fatty acid composition in other soybean tissues besides seeds, we examined the oil profile of leaves, roots and nodules for a subset of homozygous *SACPD-C* mutants and selected wild-type lines (Table 4). As previously mentioned, FN8-10 (a fast neutron induced deletion mutant) and 194D (an EMS point mutant, V211E) are derived from ‘Williams 82’. FAM94-41 is a naturally occurring mutant line selected from a cross involving cultivar Brim, which contains a spontaneous (non-induced) point mutation (D126N) in *SACPD-C*[12]. Like FN8-10 and 194D, a *SACPD-C* mutation in FAM94-41 resulted in moderately increased seed stearate (C18:0) levels compared to the reference wild-type cultivar Dare [12,28]. Stearic acid precursors (C18:0) were significantly higher and oleic acid (C18:1^Δ9cis^) precursors were significantly lower in nodules of mutant lines as compared to their wild type progenitors (Table 4). These alterations in fatty acid profile were not observed in leaf and root tissues, indicating that functional SACPD-C is not necessary in the desaturation of C18:0 to C18:1^Δ9cis^ precursors in these vegetative tissues.

**Table 4:** **Fatty acid composition of selected soybean tissues derived from hydroponically grown 4-week old *SACPD-C *** **mutant lines and wild-type lines grown in controlled growth chambers**

				^**1**^**mean % fatty acid of total ± one standard deviation**											
**Tissue**	**Genotype**	**Mutation**	**n=**	**C16:0**		**C18:0**		**C18:1**^**∆9**^		^**2**^**C18:1**^**∆11**^		**C18:2**		**C18:3**	
**Nodules**	W82	WT	5	18.8 ± 0.4	A	4.4 ± 0.7	A	3.7 ± 0.8	A	25.6 ± 2	A	31.3 ± 0.5	A	16.6 ± 0.4	A
	FN8-10	*ΔSACPD-C*	5	13.2 ± 0.5	B	11.7 ± 0.8	B	0.9 ± 0.3	C	26.4 ± 0.5	A	28.3 ± 0.7	C	19.9 ± 1.2	B
	194D	V211E	4	13.9 ± 1.3	BC	13.5 ± 1.3	B	1.3 ± 0.8	BC	20.8 ± 1.2	B	28.8 ± 0.9	C	22.1 ± 1.2	C
	Dare	WT	5	19.9 ± 0.4	A	4.1 ± 0.6	A	2.1 ± 0.8	B	26.1 ± 1.1	A	33.4 ± 1.3	B	14.7 ± 0.5	D
	FAM94-41	D126N	5	14.9 ± 0.2	C	12.6 ± 1.2	B	1.1 ± 0.4	BC	25.8 ± 1	A	31.4 ± 0.6	A	14.4 ± 0.6	D
**Roots**	W82	WT	5	26.1 ± 0.9	A	10.2 ± 2.2	A	4.9 ± 0.6	AB	1.7 ± 1.3	A	35.8 ± 1.8	A	21.6 ± 1.6	A
	FN8-10	*ΔSACPD-C*	5	26.7 ± 0.6	A	12 ± 1.7	AB	5.5 ± 1.3	A	0.9 ± 0.2	A	34.1 ± 1.5	AB	21.1 ± 1.9	A
	194D	V211E	4	29 ± 1	B	14.2 ± 1.2	B	5.3 ± 0.8	AB	1.4 ± 0.7	A	31.4 ± 0.8	B	19 ± 0.6	A
	Dare	WT	5	26.8 ± 1.3	A	11.1 ± 2.3	AB	4.1 ± 0.8	AB	0.9 ± 0.1	A	35.5 ± 2	A	21.9 ± 2.6	A
	FAM94-41	D126N	5	27.3 ± 1	AB	13.2 ± 2.1	AB	3.5 ± 0.8	B	1.2 ± 0.8	A	35 ± 2.4	AB	20.1 ± 1.9	A
**Leaves**	W82	WT	5	13.8 ± 0.5	A	5.2 ± 0.4	AB	2.1 ± 0.2	A	ND^2^		13.7 ± 0.7	A	65.4 ± 0.8	A
	FN8-10	*ΔSACPD-C*	5	14.4 ± 0.8	AB	5.3 ± 0.4	AB	2.2 ± 0.6	A	ND		14.5 ± 1.7	A	63.9 ± 2.8	A
	194D	V211E	4	14.3 ± 0.3	AB	6 ± 0.3	C	2.2 ± 0.3	A	ND		15.3 ± 1.3	A	62.5 ± 1.8	A
	Dare	WT	5	14.4 ± 0.3	AB	5.5 ± 0.2	AC	2.3 ± 0.6	A	ND		13.7 ± 1.4	A	64.4 ± 2.1	A
	FAM94-41	D126N	5	14.8 ± 0.3	B	4.9 ± 0.2	B	2.2 ± 0.4	A	ND		14.4 ± 1	A	64.1 ± 1.3	A

Height of histogram indicates mean of 4–5 samples, and bars indicate ± one standard deviation.

^1^Letters adjacent to mean + standard deviation indicates result of Tukey’s HSD test (α = 0.05); common letters indicate insignificant differences.

^2^ND = C18:1^∆11^ is derived from bacteroids/bacteria and was not detected in leaf tissues.

**Figure 8:**
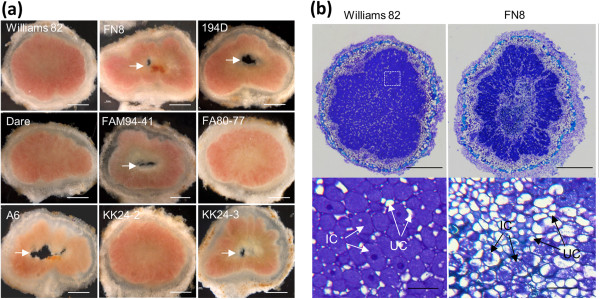
**Comparison of nodule structure formed by *B. japonicum *on wild-type and *SACPD-C *mutant soybean lines. (a)**, hand sectioned nodules showing central necrotic zones in nodules formed on *SACPD-C* mutants. Arrows indicate the central Necrotic Zones (NZ) in mutant nodules. “KK24-2” (*SACPD-C/SACPD-C*) and “KK24-3” (C298Δ/ C298Δ) are homozygote F_2:3_ plants derived from a single F_2_ plant heterozygous for the KK24 mutant allele. (*SACPD-C*/C298Δ). **(b)** Nodule sections stained with toluidine blue showing reduced bacteroids in NZ of nodules formed by mutant line FN8. IC, infected cells; UC, uninfected cells; Scale bars: 500 μm, **(a)** and upper panels in **(b)**; 50 μm, lower panels in **(b)**.

To determine if the mutations in *SACPD-C* negatively impact nodule development, we performed morphological examination of nodule sections formed by mutant and wild-type plants. Hand sections of nodules obtained from the *SACPD-C* deletion lines A6 and FN8-10, as well as the point mutant lines 194D, KK24 and FAM94-41, showed gross morphological defects which were not observed in any wild-type nodules (Figure 8a). We also examined nodules from another ‘Bay’ mutant, M23, which has increased seed oleic acid due to deletion of the *FAD2-1A* locus. Nodules of this line lacked aberrant nodule morphology (data not shown). Nodules formed by *SACPD-C* mutants showed aberrant formation of central cavities, usually accompanied by obvious discoloration (Figure 8a). Formation of central necrotic zones was observed on older nodules as early as two weeks after bacterial inoculation and was slightly more prominent in the nodules formed by the mutant line A6 (data not shown). We also examined the co-segregation of the nodule phenotype with the *SACPD-C* mutant allele derived from KK24. In a blinded experiment on progeny of a single heterozygous F_2_ plant (C298Δ/WT), we ascertained that only F_2:3_ progeny plants homozygous for the *SACPD-C* mutant allele formed aberrant nodules (Additional file 7). A minute number of degrading nodules was found in lines which inherited either heterozygosity or homozygosity for wild type alleles, and these categories were not statistically significantly different. Taken together, these segregation data and, more importantly, the occurrence of the aberrant nodule phenotype in several independent mutation events (especially three, independent point-mutation lines) provides unequivocal evidence that functional SACPD-C is required for normal nodule development in soybean. This phenotype is consistent with the high level of *SACPD-C* expression in nodules.

**Figure 9:**
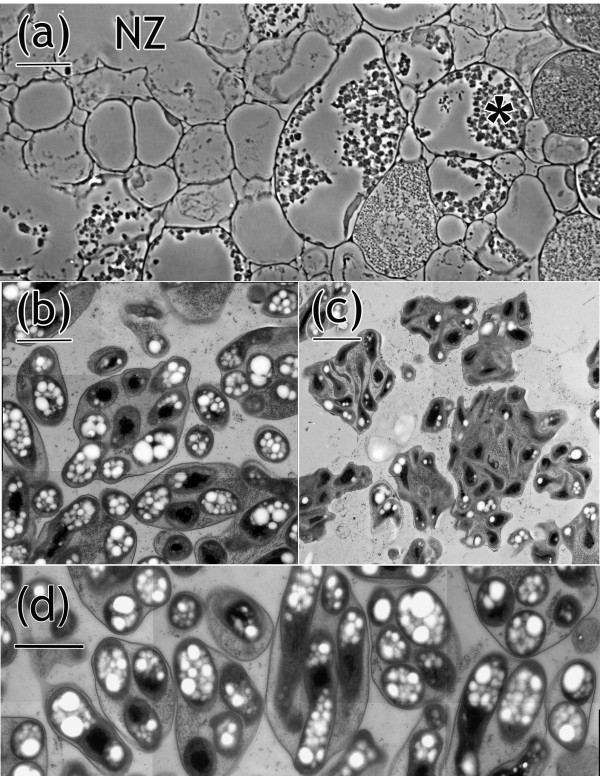
**Sectioned material of nodules from mutant line FN8 prepared by ultra-rapid freezing. (a)** Phase contrast micrograph of a thick resin section in the region bordering the central necrotic zone (NZ) from nodules of mutant line FN8. No cells were found in the NZ and those bordering it are in various stages of degradation. Cells near the NZ containing bacteroids are senescent (asterisk), with little evidence of host cytoplasm. Scale marker = 25 μm. **(b)**, **(c)** and **(d)**, Thin sections of bacteroids from a FN8 nodule. **(b)** The ultrastructure of bacteroids from host cells in the nodule periphery appears normal, indistinguishable from those in wild type nodules. **(c)** Bacteroids in cells near the NZ are senescent. **(d)** wild type bacteroids. Scale bars, 1 μm.

Nodule sections were stained with toluidine blue to further characterize the aberrant nodule development in the *SACPD-C* mutants. Microscopic examination of wild-type nodule sections showed infected nodule cells filled with toluidine blue-stained bacteroids (Figure 8b). In contrast, fewer bacteroids were observed in the necrotic regions of *SACPD-C* mutant nodules (Figure 8b). We also performed phase contrast microscopy of thick resin sections of nodules prepared for electron microscopy using ultra-rapid freezing to examine the sub-cellular detail of cells bordering the necrotic zone (Figure 9a). Cells are absent in the necrotic zone (NZ) and those bordering the NZ are in various stages of degradation. Intact cells near the NZ that contain bacteroids have little if any host cytoplasm (Figures 8b and 9a). Thin section electron micrographs of this material revealed a dichotomy of bacteroid quality. In host cells at the periphery of the nodule, bacteroid ultrastructure is indistinguishable from wild type plants (Figure 9b compared to 9d), but those in cells close to the NZ, such as marked with the asterisk in Figure 9b, are senescent (Figure 9c).

Lastly, we determined the effects of the *SACPD-C* mutations on nodule formation and symbiotic N_2_ fixation. Nodulation efficiency, measured as nodule fresh weight per plant, was not affected in the *SACPD-C* mutants (Table 5). Likewise, acetylene reduction activity, a well-established, proxy method to assay the conversion of atmospheric N_2_ into ammonium (NH_4_), showed no statistically significant difference between the wild type and *SACPD-C* mutant lines (Table 5). We did, however, note statistically significant differences between soybean cultivars ‘Williams 82’ and ‘Dare’ in nitrogenase activity but not in nodule accumulation (Table 5). The genetic basis and practical significance (if any) of these differences between the soybean cultivars are currently unknown.

**Table 5:** **Quantitation of nodule mass and nitrogen fixation in *SACPD-C *** **mutant lines and wild-type lines**

**Mean nodule fresh weight (grams) ± one standard deviation**^**1**^									
**Genotype**	**n=**	**3 weeks**		**n=**	**4 weeks**		**n=**	**5 weeks**	
W82 (WT)	11	0.25 ± 0.06	A	10	0.495 ± 0.092	A	10	0.382 ± 0.104	A
FN8-10	9	0.24 ± 0.05	A	10	0.369 ± 0.110	B	10	0.329 ± 0.075	AB
Dare (WT)	8	0.28 ± 0.06	A	10	0.275 ± 0.051	B	8	0.275 ± 0.051	B
FAM94-41	9	0.23 ± 0.06	A	9	0.328 ± 0.055	B	10	0.328 ± 0.055	AB
**Mean nitrogen fixation (μmole C**_**4**_**H**_**4 **_**gram**^**−1**^ **hr**^**−1**^**) ± one standard deviation**^**1**^									
**Genotype**	**n=**	**3 weeks**		**n=**	**4 weeks**		**n=**	**5 weeks**	
W82 (WT)	11	9.86 ± 2.67	A	10	5.27 ± 0.97	A	10	4.81 ± 1.10	A
FN8-10	9	11.28 ± 2.31	A	10	5.84 ± 2.04	A	10	6.10 ± 1.26	A
Dare (WT)	8	5.40 ± 0.72	B	10	3.52 ± 0.67	B	8	2.63 ± 0.86	B
FAM94-41	9	6.19 ± 1.21	B	9	3.23 ± 0.90	B	10	2.80 ± 1.14	B

^1^Letters adjacent to mean + standard deviation indicates result of Tukey’s HSD test (α = 0.05); common letters indicate insignificant differences between means.

## Discussion

### Clarification of the genetic mechanisms behind elevated seed stearic acid in soybean

In this work, we expanded on previous studies with soybean lines bearing elevated seed stearic acid content [12,29,30]. We analyzed radiation induced mutant lines (KK24, M25, MM106, FN8) from two independent sources (‘Bay’, ‘Williams 82’) that all had very similar elevations in stearic acid content (10 to 15% of seed oil). Deletion of the *SACPD-C* locus was reported to elevate seed stearic acid levels in A6 to ~28% [12], so we anticipated that a second locus could be mutated or deleted in these moderately elevated (10 to 15% of total oil) seed stearic acid lines.

In contrast to expectation based on the phenotype of the A6 line, we observed that deletion of the entire *SACPD-C* locus was only able to elevate seed stearic acid content to ~11 to 15% (Figure 1, Tables 1 and 3). One study reported genetic segregation of ~9% to 27% stearic acid content in a cross between the *SACPD-C* deletion line A6 and a missense *SACPD-C* mutation line FAM94-41 [28]. The overall conclusion of the researchers was that a single locus was causative, but an alternate hypothesis is that an unlinked locus is present in A6 which acts in conjunction with the *SACPD-C* deletion to elevate stearic acid content beyond the 10-15% threshold.

We evaluated the possibility that one of the two other seed expressed *SACPD* genes could be non-functional in A6. However, we found no significant differences in *SACPD-A* and *–B* mRNA accumulation (Figure 6), and no polymorphisms were identified that could affect the coding regions (Additional file 5). Collectively, these results support the hypothesis that another unidentified, non-*SACPD* locus is acting synergistically with the deletion of *SACPD-C* locus to elevate seed stearic acid levels to the extremely high levels (~28%) found in A6. The identification of the additional locus/loci will require significant population development and advancement, as detection of deletions is most successful with advanced populations which have low heterozygosity.

Prior studies indicated that sodium azide typically induces A/T → G/C transversions, but not genomic deletions [31]. We observed multiple independent polymorphisms between A6 and ‘FA8077’ (Additional file 5) within *SACPD-B*. Neither this observation nor the presence of large genomic deletions is compatible with A6 arising from sodium azide mutagenesis being performed on ‘FA 8077’. It is likely that A6 arose from a radiation-induced mutagenesis project performed on an unknown breeding line.

Inheritance of the elevated stearic trait from A6 has been reported to be associated with reduced seed yield [15] and greater than normal sensitivity to temperature stresses [32]. Although unproven, it is very likely that the seed yield reduction and temperature sensitivity of A6 may be due to the extremely large deletion affecting chromosome 14 (Figure 3). Studies with mutations allelic to A6 (Walter Fehr, personal communication 2012) but which only accumulate 10-15% stearic acid, failed to reveal a correlation with reduced protein content or reduced seed yield [15]. These deleterious effects are not apparently due to the loss of *SACPD-C*, though the deletion may contain other linked genes whose loss results in these effects.

### Applicability and limitations of CGH for analysis of deletion mutants

Although the detection of homozygous deletions was straightforward using CGH, we were unable to bridge the majority of the radiation-induced deletions by PCR with flanking PCR primers. This may be due to non-simple deletions (in contrast to the simple deletion found affecting Gm11 in KK24/M25) or due to difficulties presented by the relatively diffuse probe placement (~1100 bps on average) complicated by the ancestrally polyploid nature of the soybean genome [22]. We also noted that many such deletion borders occur in regions of the genome rich with repetitive elements. However, other approaches to detect the deletions identified by CGH can be employed, such as Southern Blot analysis or PCR amplifications to determine the presence or absence of the gene(s) of interest. All of the CNV and deletions identified for induced mutant lines (MM106, M25, KK24, FN8) identified in our studies will be publicly available on the Soybase community website (http://www.soybase.org) and will hopefully prove useful for reverse genetics approaches to annotate soybean gene function.

### SACPD-C enzymatic activity has a functional role in nodule development

The functional role of SACPD-C in converting stearic acid to oleic acid in soybean seeds is well established [12-14]. However, publicly available whole-genome soybean gene expression data show *SACPD-C* to be highly expressed in both seeds and root nodules (~5 fold higher in seeds, and ~10-fold higher in nodules) compared to *SACPD-A* or *SACPD–B* (Additional file 6). A homolog of the soybean *SAPCD-C* gene was also identified as a nodulin gene (*i.e*. gene whose expression is significant elevated during the nodulation process) in yellow lupine (*Lupinus luteus*) [33]. To obtain broader insight on the effects of *SACPD-C* mutations on plant fatty acid metabolism, we extended our fatty acid profile analysis to other plant tissues in addition to seeds. Indeed, we found altered levels of stearic and oleic acid levels in both seeds and root nodules of *SACPD-C* mutants (Tables 1, 3 and 4). In contrast, we found no consistent statistically significant differences between the fatty acid profile of either leaves or roots of *SACPD-C* mutants in comparison to parental lines (although we observed slight differences between Williams 82 and the EMS induced mutant line 194d). Our results are consistent with the “subfunctionalization” hypothesis [34-37] for restriction of *SACPD-C* function to seeds and nodules, in concordance with the *SACPD-C* expression profile. Likewise, functional redundancy among the SACPD isoforms is likely the reason for the largely unaltered fatty acid profile in leaves and roots of *SACPD-C* mutant plants.

The nodules of fast-growing annual legume species are relatively short-lived and N_2_-fixing capacity begins to decline at 3–5 weeks after infection. In determinate nodules such as those produced by soybean, senescence develops radially, starting from the center and gradually spreading toward the outside [38]. Detailed morphological evaluation of root nodules formed by *SACPD-C* mutants and wild-type parental lines indicated that mutant plants harboring any of six independent *SACPD-C* mutations showed aberrant nodule development. Mutant nodules formed central cavities surrounded by senescent cells in various stages of degradation (Figures 8 and 9), indicative of premature senescence. This nodulation phenotype was observed in the *SACPD-C* deletion lines A6, MM106 (data not shown) and FN8-10, as well as multiple, independent the point mutant lines; *i.e.* 194D (V211E), KK24 (C298Δ) and FAM94-41 (D126N). The aberrant nodule phenotype also co-segregated perfectly with homozygosity for *SACPD-C* mutant alleles encoded by KK24 in progeny of a heterozygous F_2_ plant (Additional file 7). Since multiple, independent *SACPD-C* alleles were analyzed, three of which are point mutants, we are confident that the observed nodule phenotype is due to the *SACPD-C* lesions *per se*, rather than linked co-deleted genes in A6, MM106 and FN8. The major SACPD-C isoform in soybean seeds was previously shown to have a markedly specific activity (~100-fold higher) for C18:0 precursors relative to C16:0 precursors [39]. This is also the case in nodules, as indicated by the significantly increased stearic acid (C18:0) and decreased oleic acid (C18:1) levels in *SACPD-C* mutant nodules, whereas a similar increase in C16:0 levels was not observed. The data argue for a crucial role of *SACPD-C* in nodule development with the early nodule senescence phenotype due to altered biosynthesis of stearic and oleic acid precursors in *SACPD-C* mutant nodules.

A dramatic expansion of the plant host cell membrane occurs during root nodule organogenesis and down-regulation of genes involved in membrane lipid biosynthesis and transport was recently shown to adversely affect nodule development [40-42]. Metabolomic profiling studies also showed differential accumulation of fatty acids, the key building blocks of membrane lipids, between roots and nodules, as well as between infected and uninfected root hairs [43-46]. Interestingly, oleic acid precursors, which are a direct product of SACPD enzyme action, were found to increase significantly in root hairs in response to *Bradyrhizobium japonicum* rhizobial infection [45]. The *SACPD-C* mutants showed comparable nodulation efficiency to that of wild-type plants (Table 5), indicating that a functional SACPD-C is not critical for nodule formation *per se*. However, it is unclear why alterations in fatty acid metabolism in *SACPD-C* mutant nodules can lead to premature nodule senescence.

One possibility is that changes in the ratio of saturated to unsaturated fatty acids in *SACPD-C* mutant nodules destabilizes nodule membranes, leading to premature nodule senescence. Such changes in fatty acid composition are known to affect membrane lipid fluidity in plants [47]. Moreover, biochemical and cytological studies indicate that the symbiosome membrane, *i.e*. the membrane surrounding the N_2_-fixing bacteroids, may be the first target for degradation in the nodule senescence process [38,48]. On the other hand, down-regulation of *SACPD* genes, through mutations or gene silencing, is known to trigger constitutive plant defense responses and spontaneous cell death lesions [49,50]. Altered fatty acid metabolism in *SACPD-C* mutant nodules (*e.g*., decreased oleic acid precursors) may potentially trigger plant host defense responses to restrict endosymbiont proliferation.

Under laboratory growth conditions, we did not detect a significant reduction in N_2_-fixing capability of mutant nodules compared to wild-type (Table 5). We surmise that the nitrogen fixing activity is in the peripheral nodule cells that contain healthy bacteroids (Figures 8 and 9) and in newly formed, N_2_-fixing nodules on younger roots.

Root nodule senescence is often initiated by stress conditions such as extremes of temperature, drought, pathogen or heavy metals [38]. This is consistent with the known function of polyunsaturated fatty acids in enhancing the ability of plants to tolerate environmental stresses [47,51]. Although several early nodule senescence mutants have been identified [*e.g*. [52-54]], and the value of nodulation is well documented in agriculture, relatively little is known about developmental and stress-induced nodule senescence. It is probable that SACPD-C contributes to significantly increased nodule sustainability under field conditions. Nevertheless, the full role of SACPD function in the interaction of soybean with symbiotic bacteria remains to be elucidated.

## Conclusions

Previous studies had reported that deletion of one specific soybean *Stearoyl Acyl Carrier Protein Desaturase* gene (*SACPD-C*) as solely causative for highly increased (~28%) seed stearic acid in mutant line A6. We investigated a series of five independent mutation events with moderate increases (10-15%) in seed stearic acid content, which arose from multiple genetic backgrounds and/or mutagenic agents, using comparative genome hybridization and targeted sequencing of *SACPD* genes. In contrast to expectation, all lines with moderately elevated seed stearic acid bear deletions or loss-of-function mutations affecting *SACPD-C*. A6 was found to have multiple, extremely large genomic deletions; the deletion of *SAPCD-C* in A6 consists of ~1/8 of chromosome 14 and contains at least 56 genes. Defective nodule development is unlikely to explain the yield drag seen in the A6 deletion line since all the mutants examined were altered in *SAPD-C* function but not all *SACPD-C* mutants show a reduction in yield. Therefore, it is more likely that the extraordinarily large deletion may explain A6’s extremely poor agronomic characteristics, which has hindered commercialization of high seed stearic acid lines. Another, independent locus must be deleted or mutated in line A6, which acts synergistically with the *SACPD-C* deletion to elevate seed stearic acid from ~12.5% to ~28%.

Analysis of SACPD-C in public gene expression databases revealed high expression in both developing seeds and nitrogen fixing nodules, which suggested a subfuntionalized role for *SACPD-C* in seed and nodule biology. We investigated nodules of *SACPD-C* mutant lines, from multiple genetic backgrounds and mutagenesis experiments, and found all bear dramatically altered nodule morphology and seed/nodule fatty acid profiles. Although the nodule morphological defects were not correlated with reduced nitrogen fixation under laboratory growth conditions, it is probable that SACPD-C contributes to increased nodule sustainability and/or maintenance under field conditions where plants are subjected to more pronounced environmental stresses.

## Methods

### Plant material origins

KK24, MM106 and M25 [16,17] were generously provided by Dr. Toyoaki Anai and the Legumebase seed repository. These lines were developed by X-ray mutagenesis performed on seeds of soybean cultivar ‘Bay’ [18] and identified in forward genetic screens for alteration in fatty acid composition. Seeds from lines A6 and ‘FA 8077’ [14] were kindly provided by Dr. Walter Fehr of Iowa State University. FN8 was derived from cultivar ‘Williams 82’ seed [23] dosed in 2007 with 30 Gy fast neutrons at the McClellan Nuclear Radiation Center by Dr. Kristin Bilyeu (USDA-ARS) and a portion of the total population was kindly donated to Dr. Gary Stacey. Dosed seeds were advanced three generations and were screened for a chlorotic phenotype. One of the lines selected through this screen was line FN8. Subsequent analyses showed that the chlorotic phenotype is not associated with the deletion in chromosome 14 encoding *SACPD-C* since the two traits (*i.e.* chlorosis and high stearate) segregated independently in subsequent generations. Line 194D is derived from an Ethyl Methane Sulphonate (EMS) induced ‘Williams 82’ mutant population used for TILLiNG [55]; this population was kindly donated by Kristin Bilyeu (USDA-ARS). Mutations affecting *SACPD-C* were not detected during reverse genetic analysis but later identified through a forward genetic analysis of the mutant population for fatty acid alterations.

### DNA isolation

DNA was isolated from ~20 milligrams of ground dry seed with a DNeasy kit (Qiagen, Valencia, CA, USA), according to manufacturer’s recommendations. DNA was further purified by isopropanol precipitation and resuspension when used for CGH. The purification was done by addition of ¼ volume of 5 M NaCl and mixed by inversion followed by ethanol precipitation. This preparation in 80% ethanol was chilled for 10 minutes at −20°C while equilibrating the microfuge to 4°C. Samples were spun at 14,000 × g for at least 20 min at 4°C, and then the supernatant was discarded. The pellets were washed twice by addition of ice cold 80% ethanol and a 5 minute full speed centrifugation at 4°C after each wash. Final DNA pellets were dried by SpeedVac (with no added heat) for 20 min and reconstituted in 40 uL 10 mM Tris, pH8.5.

### Comparative Genome Hybridization (CGH) for identifying genomic deletions/copy number variants

DNA fragmentation, labeling and CGH procedures were performed according to manufacturer’s recommendations (Illumina, Inc.) by staff at Mogene, Inc. (St. Louis, MO) or at the DNACore facility at the University of Missouri (FN8 only) with a custom Illumina CGH array [24]. For ‘Bay’ derived mutants and A6, a region with significantly decreased or elevated signal (relative to parental line) was only classified as a *bona fide* deletion/CNV if a minimum of three immediately adjacent probes were ≥3 standard deviations above/below the mean array signal. CNV/Deletion borders used slightly less stringent criteria and allowed by ≥2 standard deviations above/below the mean. For FN8, putative deletions or CNVs were only classified as *bona fide* if a minimum of three adjacent probes displayed log_2_ values of ≤ −1 or ≥ 1, respectively.

### Southern blot analysis

Southern blot analysis was done as previously described [56]. Briefly, soybean chromosomal DNA was isolated from young leaf tissues following routine isolation techniques. RNAse A-treated genomic DNA was digested with *HindIII* and separated on a 0.8% agarose TAE Gel. An oligonucleotide fragment at the 5′ UTR of *SACPD-C* was PCR- amplified using SACPD_C_oriF and SACPD_C_oriR primers (Additional file 3) and labeled with α^32^P-dATP (3000 Ci/mol) using the Prime-a-Gene DNA labeling system (Promega, USA). Hybridizing bands were visualized with a FujiFilm Fluorescent Imager Analyzer FLA 3000.

### Sequence analysis and qRT-PCR of SACPD genes

For three moderate stearic lines (194D, KK24, M25) and two wild type cultivars (‘Bay’, ‘FA8077’), the entire *SACPD-C* locus was PCR amplified and sequenced as previously described (Boersma *et al.*, 2012). The entire loci corresponding to *SACPD-A*, −B and –D (Glyma07g32850, Glyma02g15600 and Glyma13g08990 respectively) were also PCR amplified using Ex taq (Takara, Otsu, Shiga, Japan) in a PTC-200 thermocycler (MJ Research/Bio-Rad, Hercules, CA), under the following conditions: 95°C for an initial 5 minute denaturation, followed by 40 cycles of (95°C for 30 seconds, then 60°C for 30 seconds, and an extension step at 72°C for 1 minute per kilobase pair of expected product), using gDNA from lines KK24, M25, MM106, ‘Bay’, A6, FA 8077. PCR products were verified for correct size on 1% agarose gels and purified using Qiaquick PCR purification columns (Qiagen) before Sanger sequencing at the DNAcore facility of the University of Missouri. Sequence traces were imported into Geneious Version 5.6.5 and manually trimmed and assembled using appropriate ‘Williams 82’ genome sequences as references (ftp://ftp.jgi-psf.org/pub/compgen/phytozome/v9.0/Gmax/).

Total RNA was isolated from mid-maturation green soybean seeds (8 to 10 mm in size) from lines A6, ‘Bay’ and MM106 grown at the South Farm field location in Columbia, MO in 2012. Total RNA was DNase treated and purified as previously described [57]. A total of 400 nanograms of treated RNA was used to generate cDNA with the SMARTscribe RT kit (Clonetech, Mountain View, CA, USA) with random hexamers, and 1/20^th^ of a 20 microliter RT reaction was used in gene specific quantitative PCR with the Quatitect SYBR green PCR kit (Qiagen). A list of primers used in this work is found in Additional file 3. For each genotype/primer pair, RNA from four individual biological replicates were used for quantitation, using the deltadelta Ct method [58] with CONS6 used as a reference gene [59]. Each gene’s expression was normalized relative to the expression level using cDNA from seeds of the wild-type line ‘Bay’.

### Genotyping assays for KK24/M25 point mutant

Genotyping reactions were done using asymmetric PCR (1:5 ratio) in the presence of a Simpleprobe purchased from Fluoresentric, Inc. (Park city, UT). Primers and Simpleprobe sequence are listed in Additional file 3. Genotyping reactions were performed in a Lightcycler 480 II instrument (Roche) using the following conditions: 95°C for 5 minutes, followed by 45 cycles of 95°C for 30 seconds, 60°C for 30 seconds and 72°C for 30 seconds. Following amplification (and a one minute denaturation step at 95°C and 2 minutes at 55°C) melting curve analysis of 20 reads/°C from 55-75°C. For KK24/M25 gDNA samples, a single “melting peak” was observed at ~59°C, whereas wild type lines show a single peak at ~68°C, and heterozygotes showed both peaks (Additional file 4). Homozygous deletion lines were detected by lack of amplification product on 1% agarose gels, following symmetric PCR with KK24 primers; *SACPD-B* primers (Additional file 3) were used as a control for DNA quality.

### Nodulation and nitrogen fixation assays

Soybean plants were grown on autoclaved vermiculite and watered with half-strength plant nutrient solution [60] as needed. Bacterial inoculation was done at the time of sowing (200 μl per seed) with a commercial inoculant containing *Bradyrhizobium japonicum* (EMD Crop Biosciences, USA). Inoculated plants were grown in a growth chamber under 27°C, 70% humidity and 16-hour artificial light conditions. Nitrogen fixation activity was measured by the acetylene reduction assay [60]. Briefly, the root system was washed free of vermiculite, separated from the shoot, and put in a 22-ml vial. The vials were sealed with rubber stoppers and three ml of acetylene gas was injected into each vial. After 30 minutes of incubation, 0.5 ml of gas was drawn from each sample and injected into a Varian CP-3380 gas chromatograph equipped with a flame ionization detector. At the end of the assay, roots were taken out of the vials and nodules were separated and weighed.

### Histology and microscopy

For light microscopy, excised nodules were either hand-sectioned or were fixed for 24 hours in 50 mM sodium phosphate (pH7.0) containing 4% paraformaldehyde and 3% glutaraldehyde, and sectioned using a microtome. Hand sections were observed using a Nikon SMZ 1500 stereoscope. Microtome sections of 10 μM thick were stained with toluidine blue and photographed with an Olympus Vanox AH-3 microscope. For transmission electron microscopy, nodules were processed and imaged as previously described [61]. In brief, slices of nodule tissue were high pressure frozen (BAL-TEC HPF 010), freeze substituted for 5 days at −90°C in acetone containing 2% osmium tetroxide, warmed and embedded in Spurrs resin; thin sections stained with uranyl and lead salts were imaged in a LEO 912 AB energy filter TEM. All microscopic examinations were done on excised nodules four weeks post-inoculation. These morphological evaluations were done on nodules formed on older roots, *i.e*. on tap roots approximately 2 cm from the stem-root junction and on lateral roots formed within this region. This was done to make sure that nodules of similar developmental stage were evaluated.

### Quantification of fatty acid composition

Fatty acid composition of a portion of individual soybean (*Glycine max* (L.) Merr) seeds was examined as previously described [62]. Extraction, hydrolysis and methylation of fatty acids from nodule, leaf and root tissues were done as previously described [63] with minor modifications. Briefly, 100–300 μg of tissue samples were isolated from soybean plants four weeks post-inoculation with *B. japonicum*. To each sample, 2 ml of 5% (v/v) concentrated sulfuric acid in methanol (MeOH; freshly prepared for each use), 25 μl of BHT solution (0.2% butylated hydroxy toluene in MeOH) and 300 μl of toluene as co-solvent were added. As internal standard, heptadecanoic acid (5 mg/ml stock in toluene) was added to exactly 0.5% of dry mass of plant material. The mixture was vortexed for 30 s then heated at 90–95°C for 1.5 h. After cooling to room temperature, 1.5 ml of 0.9% NaCl (w/v) was added and FAMEs were extracted with 3 ml hexane. Lipid extracts were evaporated under nitrogen and then dissolved in 300 μl of the hexane. The FAME extracts were analyzed by GC with a flame ionization detector as previously described [64].

### Genetic and statistical analysis methods

Two mutant lines derived from ‘Bay’ (KK24, MM106) were crossed to the public soybean cultivar ‘Williams 82’. KK24 was also crossed to MM106 during the summer of 2011. Putative F_1_ and F_2_ lines were advanced in a Conviron growth chamber in the Sears plant facility located at the University of Missouri. Mineral nutrition was provided by Osmocote Plus (Scotts) per manufacturers’ recommendations. Plants were grown in 3-gallon pot (three plants per pot) using PRO-MIX (Premier Horticulture) medium and under a day/night regime of 13.5 hours day (28°C) /11.5 hours night (22°C) to produce F_2_:_3_ seed. When mature, individual F_2:3_ progeny of heterozygous F_2_ lines were harvested and chipped, with chips used for fatty acid analysis.

Fast neutron mutant line FN8 was backcrossed to ‘Williams 82’ during the summer of 2010, and BC_1_F_1_ seed were advanced in a field location in Columbia, Missouri in 2011. Individual seeds were chipped. A portion of each seed was used for fatty acid composition and another portion used for DNA isolation for genotyping assays. All individual genotype and phenotype points were correlated. Genotypic evaluation for presence/absence of *SACPD-C* for deletion lines was done using the Simpleprobe assay for KK24/M25 and a control PCR amplification for DNA quality using *SACPD-B* primers (Additional file 3).

Fatty acid phenotypic data were organized into genotypic categories based on the allelic status at *SAPCD-C* and each population was analyzed using JMP version 9 statistical software (SAS Institute). One-way ANOVA and Tukey’s HSD tests were used to determine statistically significant differences between means (α = 0.05).

## Competing interests

The authors declare they have no competing interests.

## Authors’ contributions

JDG conceived and drafted the manuscript, qRT-PCR and all seed fatty acid evaluations , and performed analyses related to A6, MM106, M25, and KK24 (field crossing and growth of plants, CGH, gDNA sequencing, molecular and genetic analysis). MGS with technical assistance from YC conceived and performed all analyses related to FN8 (field crossing and growth of plants, CGH analysis related to line ‘FN8’), Southern blot analysis, leaf and root fatty acid analyses, nodulation experiments for all lines and also assisted with drafting the manuscript. HB conducted the microscopic analysis of the nodule ultrastructure. GS supervised the reverse genetics experiments and assisted in drafting the manuscript. All authors reviewed and approved the final version.

## Supplementary Material

Additional file 1 Complete details on genomic deletions and CNV variants identified by CGH of SACPD-C mutant and their progenitors. Click here for file

Additional file 2 Comparative Genome Hybridization for Gm18 for three radiation induced mutant lines of cultivar ‘Bay’. Click here for file

Additional file 3 Oligonucleotide sequences used in this work. Click here for file

Additional file 4 Typical KK24/M25 genotyping “melt curve” SimpleProbe analysis using a Lightcycler 480 II. Click here for file

Additional file 5 **silent and intronic polymorphisms in Glym02g15600 *(SACPD-B) *** **in A6 or ‘FA8077’.** Click here for file

Additional file 6 **RNAseq expression data for four *SACPD *** **related genes in soybean tissues.** Click here for file

Additional file 7: Analysis of progeny of a single F2:3 (C298Δ/WT) plant heterozygous for the single base deletion in mutant line KK24. Click here for file
