# Supplementary figures and images for: Deletions of the SACPD-C locus elevate seed stearic acid levels but also result in fatty acid and morphological alterations in nitrogen fixing nodules
Source: BMC Plant Biol. 2014 May 27;14:143. doi: 10.1186/1471-2229-14-143 (PMC4058718; doi:10.1186/1471-2229-14-143)

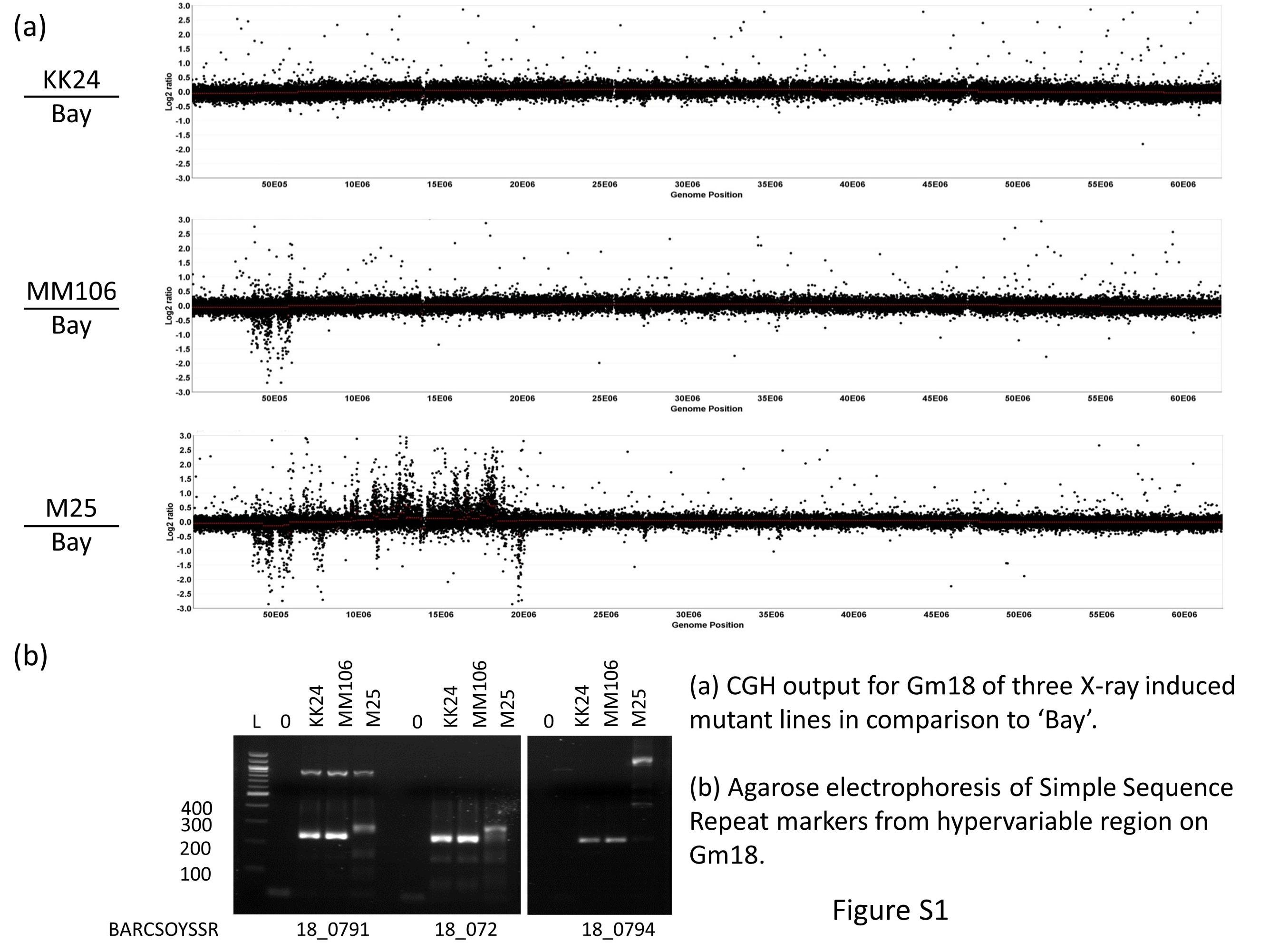

Supplement: Additional file 2 — Comparative Genome Hybridization for Gm18 for three radiation induced mutant lines of cultivar ‘Bay’. [file 1471-2229-14-143-S2.tiff]

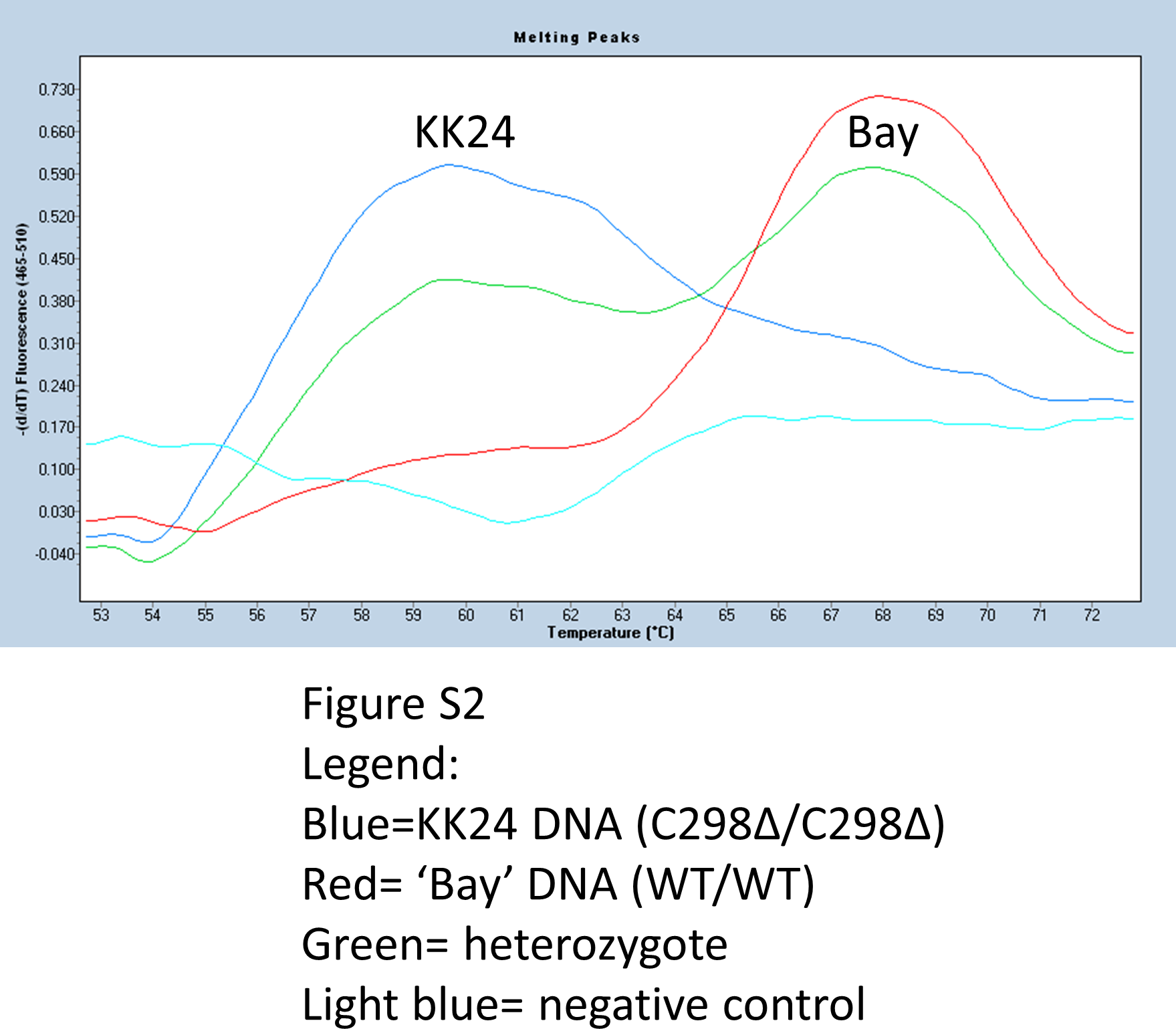

Supplement: Additional file 4 — Typical KK24/M25 genotyping “melt curve” SimpleProbe analysis using a Lightcycler 480 II. [file 1471-2229-14-143-S4.tiff]

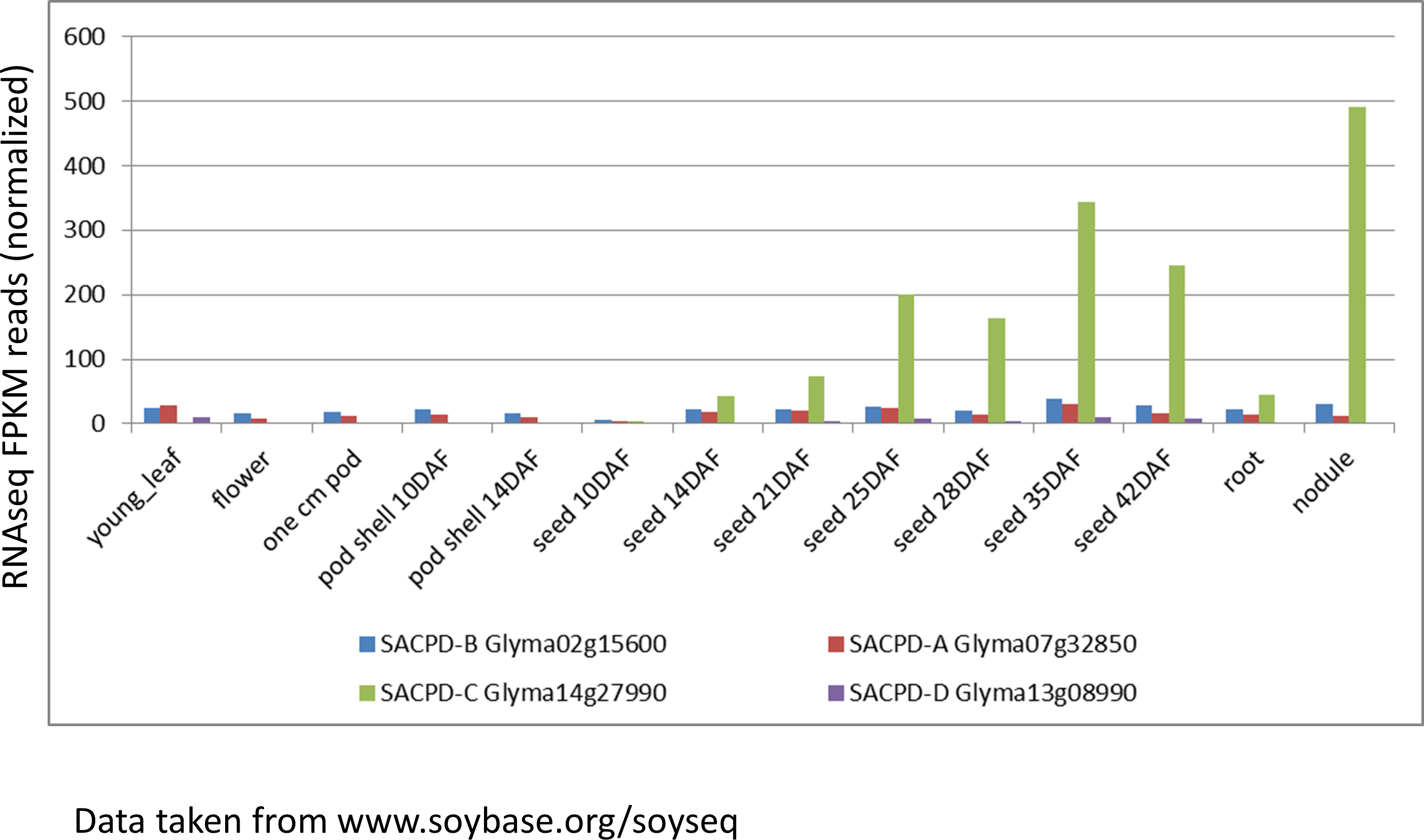

Supplement: Additional file 6 — RNAseq expression data for four SACPD related genes in soybean tissues. [file 1471-2229-14-143-S6.tiff]
